# Congenital Melanocytic Nevus of Upper Eyelid

**DOI:** 10.4103/0974-2077.79200

**Published:** 2011

**Authors:** Dinesh Singh Chauhan, Yadavalli Guruprasad

**Affiliations:** *Department of Oral and Maxillofacial Surgery, AME’S Dental College Hospital and Research Centre, Raichur, Karnataka, India*

**Keywords:** Congenital melanocytic nevus, reconstruction, upper eyelid

## Abstract

Congenital nevi are present in approximately 2–3% of neonates. These lesions are present at birth. They are characterized by pigmented lesions with regular margins, smooth or lobular surfaces and occasionally have long coarse hair. The risk of melanoma development is proportional to the size, especially if it involves over 5% of the body surface, or is > 20 cm in adolescents (large/giant congenital nevus). The risk of malignant change ranges from 5–40%. We present a case of a congenital melanocytic nevus in a six-year-old female child which was surgically treated.

## INTRODUCTION

Melanocytic nevus of the face is a unique form of congenital nevus, fortunately rare and typically hair-bearing. These lesions have a relatively high risk of becoming malignant. They develop probably between 40 days of gestation and six months *in utero*. Genetic mechanisms may account for familial aggregation. These lesions, when large, make a formidable undertaking in view of the lack of suitable donor sites and multiple procedures involved in its treatment. Melanocytic nevus of the face is a particular challenge to a plastic surgeon not only because of a very high standard of skill required but also for the patient and parental concern for cosmetic results. We present a case of melanocytic nevus in a six-year-old female child which was surgically treated.

## CASE REPORT

A six-year-old female patient was referred to the Department of Oral and Maxillofacial surgery with a chief complaint of swelling on the right upper eyelid from past two years. Examination of the patient revealed soft, well-defined swelling over the right upper eyelid [Figure 1a and b]. Swelling was small in size initially which gradually increased to a size of 6 × 3 cm and was non-tender, mobile, non-pulsatile, non-compressible and soft in consistency. The lesion involved the right upper eyelid extending on to the right medial canthus region but the normal function of both the eyelids was intact. An incisional biopsy was subsequently performed, providing a diagnosis of benign melanocytic nevus. The child was planned for surgical management under general anaesthesia. The lesion over the right upper eyelid was excised preserving a margin of 1 mm at the lid margin [Figure 2a and b] and the skin cover was provided by a full-thickness skin graft obtained from right post-auricular region which was defatted and the dermis trimmed to reduce the bulkiness of the graft so that the cosmetic as well as functional outcome would be better [Figure 3a and b]. The graft donor site was closed primarily. The graft take-up over the recipient site was 100% with excellent colour match [Figure 4a and b]. The patient was discharged on the second postoperative day in a satisfactory condition. The surgical specimen measured 6 × 2.5 cm, with irregular surface but was well circumscribed. Histopathology revealed varying proportions of melanocytic nevus cells only within the dermis [Figure 5]. Based on the microscopic observations in correlation with clinical features, a final diagnosis of congenital melanocytic nevus was made. There was no impairment of the function of the eyelids or recurrence up to six months and patient is under regular follow-up.

**Figure 1a:**
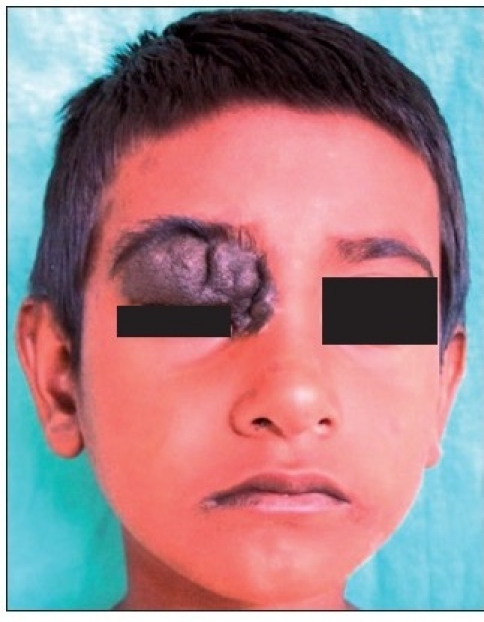
Pre-operative photograph showing swelling on the right upper eyelid

**Figure 1b:**
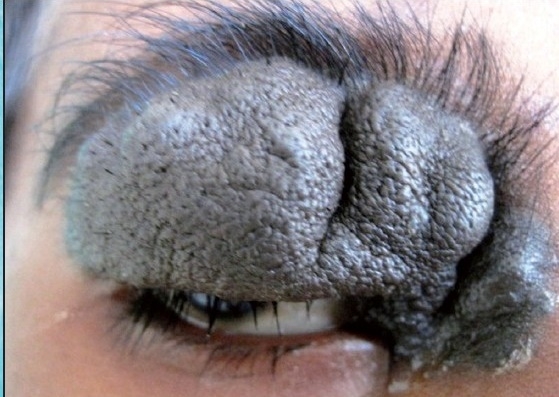
Photograph showing the size and the extent of the nevus

**Figure 2a:**
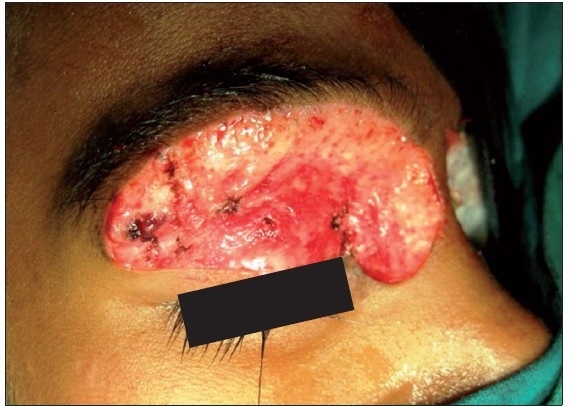
Complete surgical excision of the lesion

**Figure 2b:**
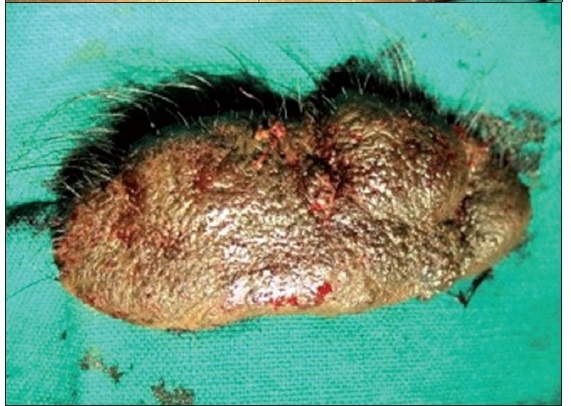
Photograph showing completely excised surgical specimen

**Figure 3a:**
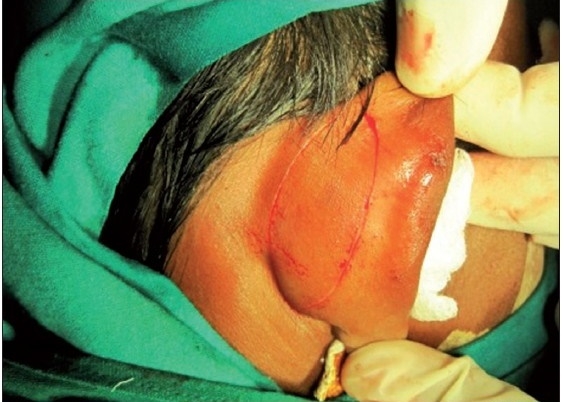
Photograph showing full-thickness graft harvested from the right post-auricular region

**Figure 3b:**
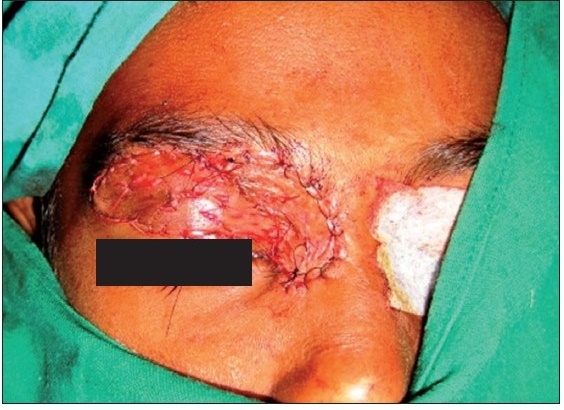
Photograph showing full-thickness graft sutured to the recipient site

**Figure 4a:**
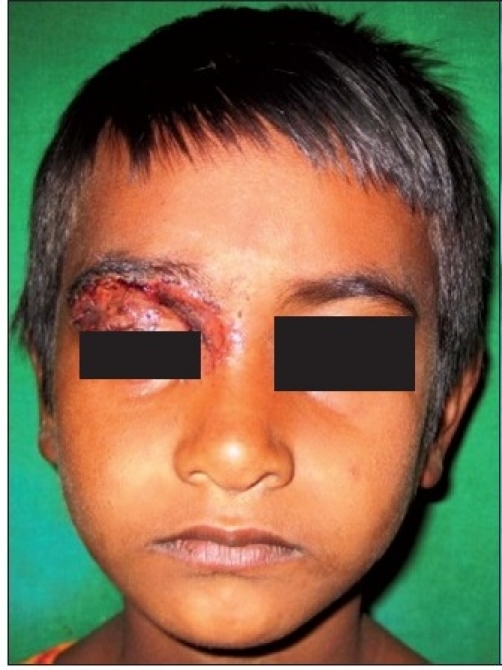
One week postoperative photograph showing the take-up of the graft

**Figure 4b:**
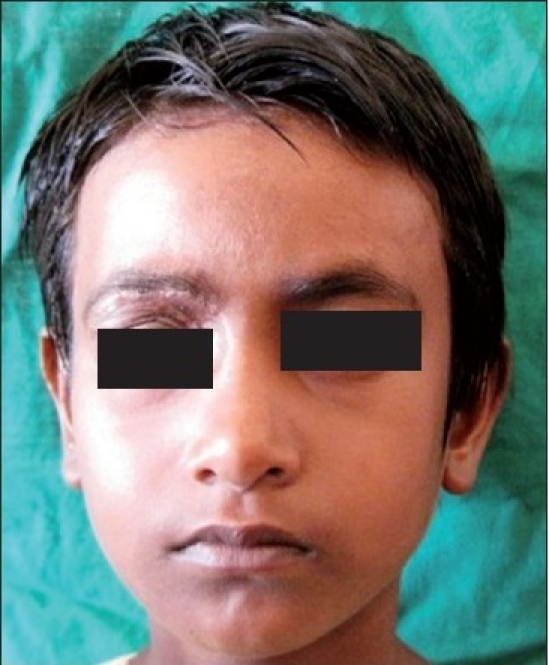
Six months postoperative photograph showing complete recovery on the right upper eyelid

**Figure 5:**
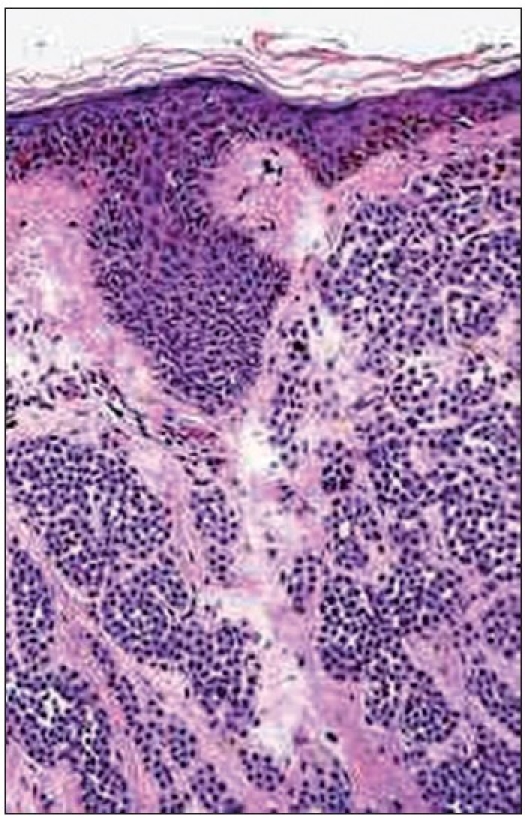
Histopathology showing varying proportions of melanocytic nevus cells only within the dermis

## DISCUSSION

Congenital pigmented nevi are present in approximately 2–3% of neonates. Fewer than 10% of these lesions are larger than 3 or 4 cm, a size cut-off below which the designation small is usually given.[1] It is now well established that congenital nevi and malignant melanoma are associated, despite the fact that the magnitude of risk of malignant transformation is still the subject of wide controversy.[2,3] Widely divergent figures range from 1.8% to up to 45%. A recent review on the subject has calculated an 8.52% incidence of melanoma developing within nevi larger than 2% of the total body surface during the first 15 years of life.[4] Despite the controversy, many clinicians agree that prophylactic excision of all giant and large hairy nevi is indicated.[5] The opinion that complete prophylactic excision of all congenital nevi should be accomplished in infancy and early childhood is well supported in the literature by the evidence that 60% of the malignancies developing in these lesions present in early childhood.[6] In one series, 10 to 20 malignant melanomas arising in congenital nevi were diagnosed before three years of age.[7] In addition, concerns about lack of parameters to predict as to which nevi are susceptible to malignant transformation, compounded with the fact that the diagnosis gets delayed and the course in case of a malignant change is invariably fatal are reasonable.[5] A recent review has concluded that an early aggressive approach to these lesions is responsible for the low risk of malignant melanoma reported in various series.[8,9] Surgical excision results in the debulking of tissue at risk for the development of malignant melanoma. Agreeing that early prophylactic excision is indicated but actually accomplishing it has posed a major management problem for most clinicians.

Large pigmented nevi of the face present a major deformity for the child and his or her parents. It is also a major challenge for the treating plastic surgeon to plan and achieve the best cosmetic results. There is complete consensus that the percentage risk of malignancy in these cases is fairly high. Complete early prophylactic excision of these lesions is warranted and should be accomplished in infancy or early childhood. The most common complications of eyelid reconstruction causing true functional impairment are ptosis, or the creation of a droopy upper eyelid and lagophthalmos, or tissue shortage preventing adequate closure.[9] Management may require multiple surgical procedures. Tissue expansion forms the most widely used modality of treatment.[10,11] Timely use of other modalities like full-thickness skin grafts and expanded flaps will decrease the number of stages of surgery while minimizing the risks of malignancy and reducing the psychological stress on the parents and the child.
